# Progress in Psychiatry

**Published:** 1900-09-01

**Authors:** 


### PROGRESS IN PSYCHIATRY.

(*Continued from page* 321.)

**Forms of Stress in the Production of Insanity.**—In a previous chapter the causation of insanity was considered under the headings of Heredity and Stress. The stable nervous system of a healthy and well-organised adult, free from hereditary taint, rarely succumbs to insanity unless the action of stresses is exceptionally great. Physical and mental overstrain, especially when occurring at times of diminished cerebral health, *e g.*, convalescence from fevers, the puerperium and lactation, want and semi-starvation, grave anæmia, &c., may readily induce melancholia, stupor, or other forms of mental disorder. The periods of puberty (fourteen to eighteen years of age), of adolescence (eighteen to twenty-five years of age), may be marked by outbreaks of insanity in predisposed subjects, and the same remark applies to the period of menopause in women (forty-five to fifty years of age). Shock, fright, violent startling, the shock and fright of a railway accident sustained without gross bodily injury, the shock of seduction childbirth and desertion in females, traumatism of the head, worry and anxiety in business or domestic life, grief and domestic calamities, all act more or less as stresses on the brain and nervous system—that part of the organism which is most sensitive and responsive to such forms of stress. A special colour may be given to the resulting psychosis by the form of stress which brings it about. It will be best to arrange under the following headings the operations of stress, some of which have been dealt with in previous articles.

A. Physical Stresses:—
(1) Toxic substances, *e.g.*, alcohol, morphia, cocaine, and other narcotics; metallic poisons, like lead.
(2) Microparasitic infections, *e.g.*, influenza, syphilis, epidemic cerebro-spinal meningitis, tuberculous meningitis, and acute delirium.
(3) Diathetic disorders, *e.g.*, myxœdema and goitre, acromegaly, diabetic and uræmic poisoning.
(4) Cerebral traumatism, *e.g.*, blows and injuries to the head, railway accidents.
(5) Cerebral overstrain and its associated insomnia, including sexual excess.
(6) Auto-intoxication from the alimentary canal.
(7) Cerebral neoplasms.

B. Evolutional Stresses, incidental to the following epochs:—
(1) Puberty (14 to 18 years of age, in both sexes).
(2) Adolescence (18 to 25 years of age).
(3) Pregnancy and the puerperium.
(4) Menopause (45 to 50 years of age).
(5) Senile involution (65 years and after, in males).

C. Moral Stresses, which on analysis are found to be complex combinations of various physical stresses:—
(1) Worry and anxiety, grief, domestic calamities, social disgrace.
(2) Mental contagion and imitation.
(3) Religious excitement.

It will be found that the stresses grouped under the first heading—viz., A, physical stresses—including in this group such potent agencies as alcohol, syphilis, infectious fevers, traumatism, &c., surpass the so-called moral stresses as factors in insanity in the proportion of about two (or more) to one. It will also be found that two or more stresses co-operate in most instances to bring about mental disorder, whether in persons previously healthy, or hereditarily possessing a taint of insanity. Where the hereditary taint is strong and well marked, slight and comparatively insignificant stresses will readily induce outbreaks of mental disorder, as, for example, in dipsomania, congenital moral and sexual perversion, paranoia, circular insanity, epileptic insanity, hysterical insanity, dementia præcox or hebephremia, melancholia with marked suicidal impulses, and recurrent mania.

The action of toxic substances in producing mental disorders is by no means perfectly understood. It is not surprising that noxious chemical substances (alcohol, morphia, cocaine, lead salts) circulating in the blood should exert their poisonous influence on the nerve-cells and nerve-fibres of the brain and spinal cord, for these elements, especially the nerve-cells of the cerebral and cerebellar cortex, basal ganglia, and bulbo-spinal nuclei, have a peculiarly abundant blood supply which amounts to double or treble of that of the white substance. The vaso-motor and circulatory disturbances produced by such substances also contribute to bring about a number of cerebral troubles, such as headaches, slight losses of consciousness, feelings of vertigo and of tinnitus aurium, flushings and throbbings about the head, and other head sensations, not to mention emotional disturbances of a vague and voluminous sort, such as malaise and depression of spirits, exhilaration, palpitation and præcordial distress, feelings of vague terror and anxiety, nervousness, general unsteadiness, &c. Finally, the local morbid changes induced in the stomach and alimentary canal by the ingestion of such substances, and the consecutive changes produced in the liver, viâ whose capillaries they enter the general circulation, have to be borne in mind. Finally, the effects produced by these toxic substances on the renal organs, the skin, and the lungs while in process of elimination further complicate matters. Moreover, many toxic agents are known to produce vascular disease (atheroma, endarteritis, periarteritis, fatty, fibroid, and hyaloid degenerations of arteries and capillaries) which slowly and secondarily bring about stases, thromboses, ischæmia, hæmorrhages, and other general or focal lesions of the nervous tissues. It will thus be seen that the action of toxic substances on the nervous system is both direct and indirect, immediate and remote in time.

